# Glial reactivity is linked to synaptic dysfunction across the aging and Alzheimer’s disease spectrum

**DOI:** 10.21203/rs.3.rs-4782732/v1

**Published:** 2024-08-12

**Authors:** Tharick Pascoal, Francieli Rohden, Pamela Ferreira, Bruna Bellaver, João Pedro Ferrari-Souza, Cristiano Aguzzoli, Carolina Soares, Sarah Abbas, Hussein Zalzale, Guilherme Povala, Firoza Lussier, Douglas Leffa, Guilherme Bauer-Negrini, Nesrine Rahmouni, Cécile Tissot, Joseph Therriault Therriault, Stijn Servaes, Jenna Stevenson, Andrea Benedet, Nicholas Ashton, Thomas Karikari, Dana Tudorascu, Henrik Zetterberg, Kaj Blennow, Eduardo Zimmer, Diogo Souza, Pedro Rosa-Neto

## Abstract

Previous studies have shown that glial and neuronal changes may trigger synaptic dysfunction in Alzheimer’s disease(AD). However, the link between glial and neuronal markers and synaptic abnormalities in the living brain is poorly understood. Here, we investigated the association between biomarkers of astrocyte and microglial reactivity and synaptic dysfunction in 478 individuals across the aging and AD spectrum from two cohorts with available CSF measures of amyloid-β(Aβ), phosphorylated tau(pTau181), astrocyte reactivity(GFAP), microglial activation(sTREM2), and synaptic biomarkers(GAP43 and neurogranin). Elevated CSF GFAP levels were linked to presynaptic and postsynaptic dysfunction, regardless of cognitive status or Aβ presence. CSF sTREM2 levels were associated with presynaptic biomarkers in cognitively unimpaired and impaired Aβ + individuals and postsynaptic biomarkers in cognitively impaired Aβ + individuals. Notably, CSF pTau181 levels mediated all associations between GFAP or sTREM2 levels and synaptic dysfunction biomarkers. These results suggest that neuronal-related synaptic biomarkers could be used in clinical trials targeting glial reactivity in AD.

Alzinova, ALZPath, Amylyx, Annexon, Apellis, Artery Therapeutics, AZTherapies, Cognito Therapeutics, CogRx, Denali, Eisai, LabCorp, Merry Life, Nervgen, Novo Nordisk, Optoceutics, Passage Bio, Pinteon Therapeutics, Prothena, Red Abbey Labs, reMYND, Roche, Samumed, Siemens Healthineers, Triplet Therapeutics, and Wave; receiving personal fees for sponsored lectures from Alzecure, Biogen, Cellectricon, Fujirebio, Lilly, Novo Nordisk, and Roche outside the submitted work; and being a cofounder of Brain Biomarker Solutions, which is a part of the GU Ventures Incubator Program outside submitted work. Dr Blennow reported having served as a consultant and at advisory boards for Acumen, ALZPath, BioArctic, Biogen, Eisai, Eli Lilly and Co, Moleac Pte Ltd, Novartis, Ono Pharma, Prothena, Roche Diagnostics, and Siemens Healthineers; having served on data monitoring committees for Julius Clinical and Novartis; having given lectures, produced educational materials, and participated in educational programs for AC Immune, Biogen, Celdara Medical, Eisai, and Roche Diagnostics; and being a cofounder of Brain Biomarker Solutions, which is a part of the GU Ventures Incubator Program, outside the submitted work. Dr Zimmer served in the SAB of Novo Nordisk, serves on SAB of Next Innovative Therapeutics (Nintx) and serves on the SAB and is a Co-founder of MASIMA. Dr Rosa-Neto served in the SAB of Novo Nordisk, Eisai, and Ely Lilly and as a Consultant in Eisai and Cerveau radiopharmaceuticals. No other disclosures were reported.

## INTRODUCTION

Alzheimer's disease (AD) is a neurodegenerative disorder clinically characterized by progressive cognitive and behavioral deficits[1]. The hallmark pathological features of AD are extracellular aggregates of amyloid-β (Aβ) plaques and intracellular inclusions of tau neurofibrillary tangles, which are associated with brain cell dysfunction and death[2]. With the advance of *in vivo* biomarkers, recent studies have indicated that both glial reactivity and synaptic dysfunction constitute an integral part of the pathophysiological cascades leading to the progression of AD[3–6]. Microglia and astrocytes are brain cells that are part of the quad-partite synapse[7], they are also involved in neuroimmune response[5,6]. Microglia are the innate immune system's primary cells in the central nervous system, which might overexpress proteins associated with AD progression, such as the triggering receptor expressed on myeloid cells 2 (TREM2)[8]. Astrocytes are cells involved in multiple physiological processes that become reactive in response to the Aβ or tau aggregation, undergoing molecular, morphological, and functional changes, including the increased expression of glial fibrillary acidic protein (GFAP)[9–11]. The importance of understanding the interplay between quad-partite synapses and classical AD proteinopathies (i.e., Aβ and tau) has been evidenced by a growing body of experimental and biomarker studies[12–16].

The onset of synaptic dysfunction seems to precede neuronal loss and exhibits a stronger correlation with cognitive decline compared to Aβ plaques or tangles alone[3,4]. Reduced and compromised synaptic functionality is a key feature of individuals with cognitive impairment[17]. The process of acquiring and consolidating information in the human brain fundamentally depends on functional synapses, which are the primary orchestrators of neuronal communication[18,19]. The presynaptic biomarker growth-associated protein 43 (GAP43) appears to play a role in cognition and its levels were found to be increased in the CSF symptomatic AD patients[20,21]. Similarly, the postsynaptic marker neurogranin (Ng) is involved in facilitating long-term synaptic potentiation[22]. Elevated CSF Ng levels have been suggested as a biomarker of postsynaptic degeneration in AD[22,23]. If well validated in the context of the pathophysiological progression of AD, synaptic biomarkers can potentially be used to monitor disease progression and treatment effects[24]. It is known from the experimental literature that glial cells play fundamental roles in these processes, reducing neurotransmitters in the synaptic cleft to a sufficient level, preserving physiological balance, and releasing neuroactive agents that modulate synaptogenesis[19]. Based on this, we hypothesize that phosphorylated tau, may potentially trigger inflammatory responses[25,26] and mediate interactions between glial cells and pre- and post-synaptic neuronal biomarkers.

This study aims to fill a gap in the existing literature by examining the existence of a link between glial reactivity and synaptic dysfunction biomarkers throughout the progression of AD. The insights derived from this study could be fundamental in defining therapeutic strategies focusing on glial cell reactivity, potentially using synaptic biomarkers as secondary endpoints.

## RESULTS

We assessed 107 individuals from the TRIAD cohort, of which 42 were CU Aβ-, 22 CU Aβ+, and 43 were CI Aβ+; and 373 individuals from ADNI, of which 59 were CU Aβ-, 54 were CU Aβ+, and 260 were CI Aβ + . The sample characteristics are described in **Table 1**.

Astrocyte reactivity is associated with synaptic markers independently of Aβ.

We observed significant associations between CSF GFAP and both GAP43 and Ng across all groups in the TRIAD cohort [CU Aβ- GAP43: β = 0.37, 95% CI = 0.04–0.71, p = 0.03, Ng: β = 0.36, 95% CI = 0.01–0.70, p = 0.04 (Fig. 1a,d); CU Aβ + GAP43: β = 0.93, 95% CI = 0.41–1.45, p = 0.001, Ng: β = 0.38, 95% CI = 0.12–0.64, p = 0.007 (Fig. 1b,e); CI Aβ + GAP43: β = 0.33, 95% CI = 0.02–0.34, p = 0.04, Ng: β = 0.38, 95% CI = 0.02–0.74, p = 0.04 (Fig. 1c,f)]. These parameters were not assessed in the ADNI cohort because it did not have available measures of CSF GFAP in the studied population.

Microglial activation is associated with synaptic markers only in the presence of Aβ.

In both TRIAD and ADNI cohorts, CSF sTREM2 was significantly associated with GAP43 only in Aβ + individuals, independently of cognitive status (CU Aβ-: TRIAD β = 0.19, 95% CI=-0.07–0.07, p = 0.17; ADNI: β = 0.22, 95% CI=-0.01-0.44, p = 0.06, Fig. 2a,d; CU Aβ+: TRIAD β = 0.58, 95% CI=-0.39-0.72, p = 0.02; ADNI: β = 0.55, 95% CI = 0.32–0.79, p < 0.0001, Fig. 2b,e; CI Aβ+: TRIAD: β = 0.38, 95% CI = 0.01–0.75, p = 0.01, ADNI: β = 0.39, 95% CI = 0.27–0.51, p < 0.0001, Fig. 2c,f). In both TRIAD and ADNI, CSF sTREM2 was associated with Ng only in CI Aβ + individuals (CU Aβ-: TRIAD β = 0.12, 95% CI=-0.17-0.34, p = 0.34; ADNI β = 0.11, 95% CI=-0.15-0.37, p = 0.38, Fig. 2g,j; CU Aβ+: TRIAD β = 0.29, 95% CI=-0.13-0.72, p = 0.16; ADNI β = 0.18, 95% CI=-0.65-0.28 p = 0.41, Fig. 2h,k; CI Aβ+: TRIAD β = 0.36, 95% CI = 0.07–0.66 p = 0.01, ADNI β = 0.20, 95% CI=-0.01-0.39 p = 0.04, Fig. 2i,l).

## Synaptic dysfunction as a function of glial reactivity across the AD continuum

Lowess curves corroborate previous results by demonstrating a progressive increase in pre- and post-synaptic markers as a function of astrocyte reactivity independently of Aβ (Fig. 3a), while synaptic markers increased as a function of microglial reactivity only in CU Aβ + and CI Aβ+ (Fig. 3b). When analyzing synaptic biomarkers as a function of GFAP, we observed a steeper increase in CU Aβ + cases compared to CU Aβ- and CI Aβ + . Conversely, regarding sTREM2, we find similar increases in synaptic biomarkers in both CU Aβ + and CI Aβ + .

PTau181 mediates the association between glial reactivity and synaptic dysfunction.

In the TRIAD cohort, we found that the influence of GFAP on both pre- and post-synaptic markers occurred through CSF pTau181 in all groups. Specifically, mediation analyses revealed that CSF pTau181 fully mediated the effects of CSF GFAP on CSF GAP43 in CU Aβ- (direct pathway, $p = 0.87$; indirect pathway, $p < 0.0001$), in CU Aβ+ (direct pathway, $p = 0.23$; indirect pathway, $p = 0.03$), and CI Aβ+ (direct pathway, $p = 0.94$; indirect pathway, $p < 0.0001$; Fig. 4a-c). Similarly, pTau181 also fully mediated the effects of CSF GFAP on CSF Ng in CU Aβ- (direct pathway, $p = 0.82$; indirect pathway, $p = 0.004$), in CU Aβ+ (direct pathway, $p = 0.12$; indirect pathway, $p < 0.0001$), and CI Aβ+ (direct pathway, $p = 0.47$; indirect pathway, $p < 0.0001$; Fig. 4d-f). We observed, in TRIAD and ADNI combined, that pTau181 acted as a mediator in the association of sTREM2 with the presynaptic marker GAP43 in the presence of Aβ positivity and with the postsynaptic marker Ng in the additional presence of cognitive impairment. Specifically, mediation analyses showed that CSF pTau181 partially mediated the effects of sTREM2 on CSF GAP43 in CU Aβ+ (direct pathway, $p = 0.05$; indirect pathway, $p < 0.0001$) and CI Aβ+ (direct pathway, $p < 0.0001$; indirect pathway, $p < 0.0001$), but not in CU Aβ- (direct pathway, $p = 0.78$; indirect pathway, $p = 0.98$; (Fig. 4g-i). In addition, CSF pTau181 partially mediated the effects of sTREM2 on CSF Ng in CI Aβ+ (direct pathway, $p = 0.80$; indirect pathway, $p = 0.004$), but not in CU Aβ- (direct pathway, $p = 0.24$; indirect pathway, $p = 0.52$) and CU Aβ+ (direct pathway, $p = 0.32$; indirect pathway, $p = 0.06$; Fig. 4j-l).

## DISCUSSION

This study indicates a differential association between glial responses and synaptic abnormalities in AD. Our results support that astrocyte reactivity is closely associated with pre- and post-synaptic dysfunction in patients across the aging and AD continuum. By contrast, the association between microglial activation and synaptic biomarkers was dependent on the presence of Aβ positive (Fig. 5). We also found that CSF pTau181 mediated the association between glial reactivity and synaptic dysfunction in living humans.

CSF GFAP was associated with synaptic biomarkers in CU and CI individuals independently of Aβ pathology. These results suggest early astrocyte reactivity triggering synapse damage independently of the emergence of Aβ aggregates. This observation aligns with a recent study suggesting that GFAP

abnormalities precede Aβ deposition[35]. Furthermore, previous studies have shown an independent link between astrocyte reactivity and aging[36] and neurodegeneration[37]. In addition, GFAP has been associated with synaptic and neuronal degeneration in other brain conditions that are not characterized by the presence of Aβ pathology, such as Parkinson's disease[38] and traumatic brain injury[39]. It is important to highlight that although our results support that GFAP imposes synaptic dysfunction independently of Aβ, it also contributed to synaptic dysfunction in symptomatic AD individuals[40]. Together, these findings support the notion that astrocyte reactivity is linked to a disruption of synaptic homeostasis contributing to aging and AD progression.

CSF sTREM2 was associated with synaptic biomarkers only in the presence of Aβ pathology. Our results are consistent with previous literature suggesting that Aβ pathology activates microglia in AD[41]. These studies suggest that microglial activation-related immunoresponses might potentiate Aβ aggregation[42–45] and that Aβ pathology and microglial reactivity synergistically interact to determine cognitive deterioration in individuals across the AD spectrum[16]. Taken together, these results highlight synaptic biomarkers as potential secondary endpoints for clinical trials testing novel therapeutic strategies aimed at mitigating microglial dysfunction in patients with underlying AD pathology.

We found that CSF pTau mediates the association between glial reactivity and synaptic dysfunction. Previous studies have shown that pTau, can potentially trigger inflammatory responses and be detected by microglia[25,26]. This process appears to mediate the link between glial reactivity and synaptic dysfunction. In this context, our findings corroborate previous research suggesting a close connection between tau secretion, microglial activation, and synaptic damage. Specifically, studies have shown that synaptic dysfunction precedes neurodegeneration and occurs concurrently with elevated CSF pTau in preclinical AD[43–46]. The density of neurofibrillary tangles is associated with synaptic dysfunction, leading to cognitive decline[43,44]. Tau protein binds to synaptic vesicle proteins, accumulating excessively in presynaptic terminals, causing synaptic dysfunction[43]. Although evidence demonstrates independent associations between glia, tau, and synaptic damage, it is not yet understood how these pathologies interact with each other to determine pathological progression[45–47]. In this context, our results propose a model where tau phosphorylation mediates the deleterious effects of glial reactivity on synaptic dysfunction. These findings suggest that reducing tau phosphorylation may potentially mitigate the effects of glial reactivity on synapses.

A strength of our study is that all results were replicated in two independent and well-characterized cohorts. It is important to acknowledge the limitations of our study. A population-based longitudinal study with multiple time points would be desirable to provide a more comprehensive understanding of the temporal dynamics and sequential relationships between biomarkers. Future mechanistic studies are needed to further corroborate our clinical findings. Our group of participants is mainly composed of White individuals, emphasizing the importance of replicating our findings using a more diverse sample of individuals to increase the external validity of results for the more diverse world population.

In conclusion, our findings support a link between glial reactivity and synaptic dysfunction in living humans, which appears to be explained by pathological phosphorylation of tau. While astrocyte reactivity seems to be a partially independent phenomenon leading to synaptic dysfunction in aging and AD, the effects of microglial activation on synaptic function are determined by the emergence of Aβ pathology. These results suggest that synaptic biomarkers hold potential as secondary endpoints for clinical trials targeting glial reactivity in aging and AD.

## MATERIALS AND METHODS

### Participants

We included participants from two cohorts: The Translational Biomarkers in Aging and Dementia (TRIAD) cohort at McGill University, Canada (https://triad.tnlmcgill.com) and the Alzheimer's Disease Neuroimaging Initiative (ADNI) database (https://adni.loni.usc.edu/). For TRIAD participants, the exclusion criteria were inability to speak English or French, inadequate visual and auditory capacities for neuropsychologic assessment, active substance abuse, major surgery, recent head trauma, medical contraindication for positron emission tomography (PET) or magnetic resonance imaging (MRI), and neurological, psychiatric, or systemic comorbidities that were not adequately treated with a stable medication regimen[13]. ADNI inclusion and exclusion criteria are available at https://adni.loni.usc.edu/. Recruited participants were between the ages of 55 and 90 years, completed at least six years of education, were fluent in Spanish or English, had a Hachinski ischemic score less than or equal to four, and had screening/baseline MRI scans without evidence of infection, infarction, or other focal lesions (individuals with multiple lacunes or lacunes in a critical memory structure were excluded). We assessed a total of 480 individuals: 107 from the TRIAD cohort [64 cognitively unimpaired (CU), 43 cognitively impaired (CI)], and 373 from the ADNI cohort [113 CU, and 260 CI]. We included all participants from these cohorts who were 50 years or older, who had available CSF GFAP, soluble TREM2 (sTREM2), GAP43, Ng, Aβ42, Aβ42/40, and tau protein phosphorylated at threonine 181 (pTau181) values. CU and CI were defined as described elsewhere[27]: CU individuals had no memory complaints and a Mini-Mental State Examination (MMSE) score between 24 and 30, while CI individuals (including patients with mild cognitive impairment (MCI) and dementia) had an MMSE score below 24. We defined Aβ positivity using a published validated cutoff for CSF Aβ42 abnormality for each cohort[28,29]. The studies were approved by institutional review boards of all participating centers, and written informed consent was obtained from all participants or their authorized representatives were applicable.

### CSF measurement

In the TRIAD cohort, CSF data was analyzed at the University of Gothenburg, Sweden blinded to participant clinical information. CSF GFAP levels were quantified on the Simoa HD-X (Quanterix) using the commercial single-plex assay (No. 102336)[30]; CSF sTREM2 concentration was measured using a Meso-Scale Discovery assay, as described previously[30]; CSF pTau181 and Aβ42/40 were quantified by LUMIPULSE G1200 (Fujirebio)[28]; synaptic biomarkers, GAP43 and Ng were measured using *in-house*

immunoassays developed at the University of Gothenburg[31]. The ADNI cohort procedure manual describes the CSF collection process (http://www.adni-info.org/). CSF sTREM2 measurements were performed using ELISA by the laboratory of C. Haass at the DZNE Munich as described previously[32]; CSF Aβ42 and CSF pTau181 were quantified using the Elecsys assays (Roche Diagnostics)[29]; CSF GAP-43 quantification was performed using an in-house ELISA method at the Clinical Neurochemistry Laboratory at the Sahlgrenska University Hospital (Mölndal, Sweden)[33]; CSF Ng concentration was measured by electrochemiluminescence as described previously[21,32].

## Statistical analysis

The values were standardized using z-scores centered on the mean within each cohort. Continuous variables were evaluated for differences between groups using analysis of variance (ANOVA) with post hoc Tukey comparison. For categorical or ordinal variables, the Kruskal-Wallis test was used followed by the Mann-Whitney U post hoc test. We examined the associations between variables using linear regression models adjusted for sex and age. We used the locally weighted scatterplot smoothing (Lowess) method involving 2,000 iterations with a smoothing span of 1.00 to model biomarker trajectories[13]. The mediation analysis was conducted for the observation of both direct and indirect effects. Here, the direct effect refers to the relationship between glial markers and synaptic markers, while the indirect effect, mediated by pTau181, captured the association between these variables. The significance of the mediation was assessed by calculating bias-corrected 95% confidence intervals using bootstrapping with 500 resamples[34]. Biomarker values were standardized using mean-centered z-scores within each cohort, adjusted for sex and age, allowing integration of the TRIAD and ADNI data for mediation analyses, in addition, the model was adjusted with the cohort factor. Biomarker values were standardized using z-scores centered on the mean within each cohort enabling the integration of TRIAD and ADNI cohorts for mediation analyses. $P < 0.05$ indicated a significant difference. Statistical analyses were conducted using the R software (version 4.0.2, http://www.r-project.org/).

### Declarations

## Conflict of Interest

Dr Therriault has served as a paid consultant for the Neurotorium educational platform and for Alzheon. Dr Ashton received payment for lectures from Biogen, BioArtic, Eli-Lily, and Quanterix. Dr. Karikari reported receiving grants from the National Institutes of Health and receiving personal fees from the University of Wisconsin–Madison and the University of Pennsylvania outside the submitted work; in addition, Dr Karikari had a patent for WO2020193500A1 issued. Dr Zetterberg reported receiving personal fees from Abbvie, Acumen, Alector, Alzinova, ALZPath, Amylyx, Annexon, Apellis, Artery Therapeutics, AZTherapies, Cognito Therapeutics, CogRx, Denali, Eisai, LabCorp, Merry Life, Nervgen, Novo Nordisk, Optoceutics, Passage Bio, Pinteon Therapeutics, Prothena, Red Abbey Labs, reMYND, Roche, Samumed, Siemens Healthineers, Triplet Therapeutics, and Wave; receiving personal fees for sponsored lectures from Alzecure, Biogen, Cellectricon, Fujirebio, Lilly, Novo Nordisk, and Roche outside the submitted work; and being a cofounder of Brain Biomarker Solutions, which is a part of the GU Ventures Incubator Program outside submitted work. Dr Blennow reported having served as a consultant and at advisory boards for Acumen, ALZPath, BioArctic, Biogen, Eisai, Eli Lilly and Co, Moleac Pte Ltd, Novartis, Ono Pharma, Prothena, Roche Diagnostics, and Siemens Healthineers; having served on data monitoring committees for Julius Clinical and Novartis; having given lectures, produced educational materials, and participated in educational programs for AC Immune, Biogen, Celdara Medical, Eisai, and Roche Diagnostics; and being a cofounder of Brain Biomarker Solutions, which is a part of the GU Ventures Incubator Program, outside the submitted work. Dr Zimmer served in the SAB of Novo Nordisk, serves on SAB of Next Innovative Therapeutics (Nintx) and serves on the SAB and is a Co-founder of MASIMA. Dr Rosa-Neto served in the SAB of Novo Nordisk, Eisai, and Ely Lilly and as a Consultant in Eisai and Cerveau radiopharmaceuticals. No other disclosures were reported.

## Acknowledgments

Dr. Pascoal is supported by the NIA (5R01AG075336, 5R01AG073267). Dr. Rohden receives financial support from Conselho Nacional de Desenvolvimento Científico e Tecnológico (CNPq) (102380/2022-8). Dr. Ferreira is supported by the Alzheimer’s Association (AARFD-22-923814). Dr. Bellaver is supported by the Alzheimer’s Association (grant No. AARFD-22-974627) and the National Institute of Aging (5 P01 AG025204-17). Dr. Povala is supported by the Alzheimer’s Association (24AARFD-1243899). Ms. Soares has received funding from Coordenação de Aperfeiçoamento de Pessoal de Nível Superior (CAPES) [88887.696202/2022-00] and Conselho Nacional de Desenvolvimento Científico e Tecnológico (CNPq) [141357/2020-7]. Dr. Bauer-Negrini is supported by the Alzheimer’s Association (AARFD-23-1150249). Dr. Schaffer Aguzzoli is supported by the Global Brain Health Institute, Alzheimer’s Association, and Alzheimer’s Society (grant No. GBHI ALZ UK-23-971089), and Alzheimer’s Association (grant No. 24AACSF-1200375). Mr. Ferrari-Souza is supported by Conselho Nacional de Desenvolvimento Científico e Tecnológico (No. 200691/2021-0). Dr. Therriault is funded by the Colin J Adair Family Foundation Scholarship. Dr. Karikari receives financial support from the NIH (R01 AG083874 and U24 AG082930), Swedish Research Council (Vetenskåpradet; #2021–03244), Alzheimer’s Association (#AARF21-850325), Swedish Alzheimer Foundation (Alzheimerfonden), Aina (Ann) Wallströms and Mary-Ann Sjöbloms stiftelsen, Emil och Wera Cornells stiftelsen. Dr. Zetterberg is a Wallenberg Scholar and a Distinguished Professor at the Swedish Research Council supported by grants from the Swedish Research Council (#2023 – 00356; #2022 – 01018 and #2019–02397), the European Union’s Horizon Europe research and innovation programme under grant agreement No 101053962, Swedish State Support for Clinical Research (#ALFGBG-71320), the Alzheimer Drug Discovery Foundation (ADDF), USA (#201809–2016862), the AD Strategic Fund and the Alzheimer's Association (#ADSF-21-831376-C, #ADSF-21-831381-C, #ADSF-21-831377-C, and #ADSF-24-1284328-C), the Bluefield Project, Cure Alzheimer’s Fund, the Olav Thon Foundation, the Erling-Persson Family Foundation, Familjen Rönströms Stiftelse, Stiftelsen för Gamla Tjänarinnor, Hjärnfonden, Sweden (#FO2022-0270), the European Union’s Horizon 2020 research and innovation programme under the Marie Skłodowska-Curie grant agreement

No 860197 (MIRIADE), the European Union Joint Programme – Neurodegenerative Disease Research (JPND2021-00694), the National Institute for Health and Care Research University College London Hospitals Biomedical Research Centre, and the UK Dementia Research Institute at UCL (UKDRI-1003). Dr. Blennow is supported by the Swedish Research Council (2017 – 00915), the Alzheimer Drug Discovery Foundation (RDAPB-201809-2016615), the Swedish Alzheimer Foundation (AF-742881), Hjärnfonden (FO2017-0243), the Swedish state under the agreement between the Swedish government and the County Councils, the ALF-agreement (ALFGBG-715986), the European Union Joint Program for Neurodegenerative Disorders (JPND2019-466-236), the NIH (1R01AG068398-01), and the Alzheimer's Association 2021 Zenith Award (ZEN-21-848495). Dr. Zimmer receives financial support from CNPq [312410/20182; 435642/2018-9; 312306/2021-0; 409066/2022-2], ARD/FAPERGS [21/2551-0000673-0], AA [AARGD-21-850670], CNPQ/FAPERGS/PRONEX [16/2551-0000475-7], the Brazilian National Institute of Science and Technology in Excitotoxicity and Neuroprotection [465671/2014-4], Instituto Serrapilheira [Serra-1912-31365], and National Academy of Neuropsychology [ALZ-NAN-22-928381]. Dr. Souza is supported by CNPQ/INCT [465671/2014-4], CNPQ/ FAPERGS/PRONEX [16/2551- 0000475-7], and FAPERGS [19/2551-0000700-0]. Dr. Rosa-Neto is funded by Fonds de Recherche du Québec–Santé (Chercheur Boursier, PR-N and 2020-VICO-279314) and CIHR-CCNA. TRIAD cohort was supported by grants MOP-11-51-31 and RFN 152985, 159815, 162303 from the Canadian Institutes of Health Research (CIHR); MOP-11-51-31 from the Canadian Consortium of Neurodegeneration and Aging (CCNA); the Weston Brain Institute; and grants NIRG-12-92-92090 and NIRP-12-259245 from the Alzheimer Association.

## Tables

Table 1. Demographics and key characteristics of participants.

| TRIAD | CU Aβ- (N = 42) | CU Aβ+ (N = 22) | CI Aβ+ (N = 43) |
|---|---|---|---|
| Sex (N, F %) | 26 (61.9%) | 14 (63.6%) | 23 (53.5%) [NS] |
| Age (Years) | 69.9 (6.68) | 72.1 (3.94) | 67.6 (7.53) [ac] |
| MMSE (score) | 29.0 (0.96) | 29.1 (0.92) | 25.3 (4.62) [a,b] |
| Education (Years) | 15.5 (3.54) | 14.0 (3.33) | 15.3 (3.10) [NS] |
| APOE ε4 carriers (No. %) | 9 (21.4%) | 8 (36.4%) | 21 (48.8%) [a,b,c] |
| CSF GFAP (z-score) | 0.03 (0.75) | 0.19 (0.79) | 0.55 (0.90) [a] |
| CSF sTREM2* (z-score) | 0.41 (1.09) | 0.02 (0.87) | 0.08 (0.88) [NS] |
| CSF GAP43 (z-score) | -0.31 (0.70) | 0.36 (1.11) | 0.53 (0.97) [a] |
| CSF Ng (z-score) | -0.35 (0.74) | 0.27 (0.91) | 0.61 (1.12) [a] |
| CSF Aβ42 (z-score) | 0.89 (0.84) | -0.42 (0.75) | -0.80 (0.52) [a,c] |
| CSF pTau181 (z-score) | -0.53 (0.21) | 0.17 (0.96) | 0.93 (1.10) [a,b] |
| ADNI | CU Aβ− (N = 59) | CU Aβ+ (N = 54) | CI Aβ+ (N = 260) |
| Sex (N, F %) | 29 (49.2%) | 26 (48.1%) | 96 (36.9%) [a,b] |
| Age (Years) | 71.3 (5.95) | 74.6 (5.79) | 72.9 (7.69) [NS] |
| MMSE (score) | 29.3 (0.95) | 28.9 (1.37) | 25.0 (2.93) [a,b] |
| Education (Years) | 17.1 (2.31) | 16.5 (2.28) | 16.0 (2.77) [a] |
| APOE ε4 carriers (N, No. %) | 15 (25.4%) | 21 (38.9%) | 124 (47.7%) [a,b,c] |
| CSF sTREM2 (z-score) | -0.18 (0.78) | -0.16 (0.89) | 0.02 (1.03) [NS] |
| CSF GAP43 (z-score) | -0.31 (0.63) | -0.33 (0.89) | 0.19 (1.07) [a,b] |
| CSF Ng* (z-score) | -0.52 (0.63) | -0.19 (0.99) | 0.20 (1.03) [a] |
| CSF Aβ42 (z-score) | 1.41 (0.60) | -0.38 (0.48) | -0.63 (0.48) [a,c] |
| CSF pTau181 (z-score) | -0.59 (0.58) | -0.37 (0.71) | 0.34 (1.01) [a,b,c] |

Continuous variables are presented as mean (SD). CU = cognitively unimpaired, CI = cognitively impaired, MMSE = Mini-Mental State Examination, APOE ε4 = Apolipoprotein E ε4, CSF = cerebrospinal fluid, PET =

positron emission tomography, Aβ = amyloid-β, SD = standard deviation. Missing APOEε4 carriership data in TRIAD: CU Aβ− = 1 (2.4%); CI Aβ+ = 4 (9.3%) and ADNI: Missing: CU Aβ+ = 5 (9.3%); CI Aβ+ = 54(20.8%). In the sTREM2 analysis of CSF in the CU Aβ− group (n = 38), CU Aβ + group (N = 23), and CI Aβ + group (N = 45), this small difference in "N" did not influence the final results. *CSF Ng in ADNI CU Aβ− N = 25, CU Aβ + N = 19 and CI Aβ + N = 121. [a]CI Aβ + compared to CU Aβ-, [b]CI Aβ + compared to CU Aβ + and [c]CU Aβ- compared to CI Aβ+, $p < 0.05$ = Significant, NS = Not Significant.

## Figures

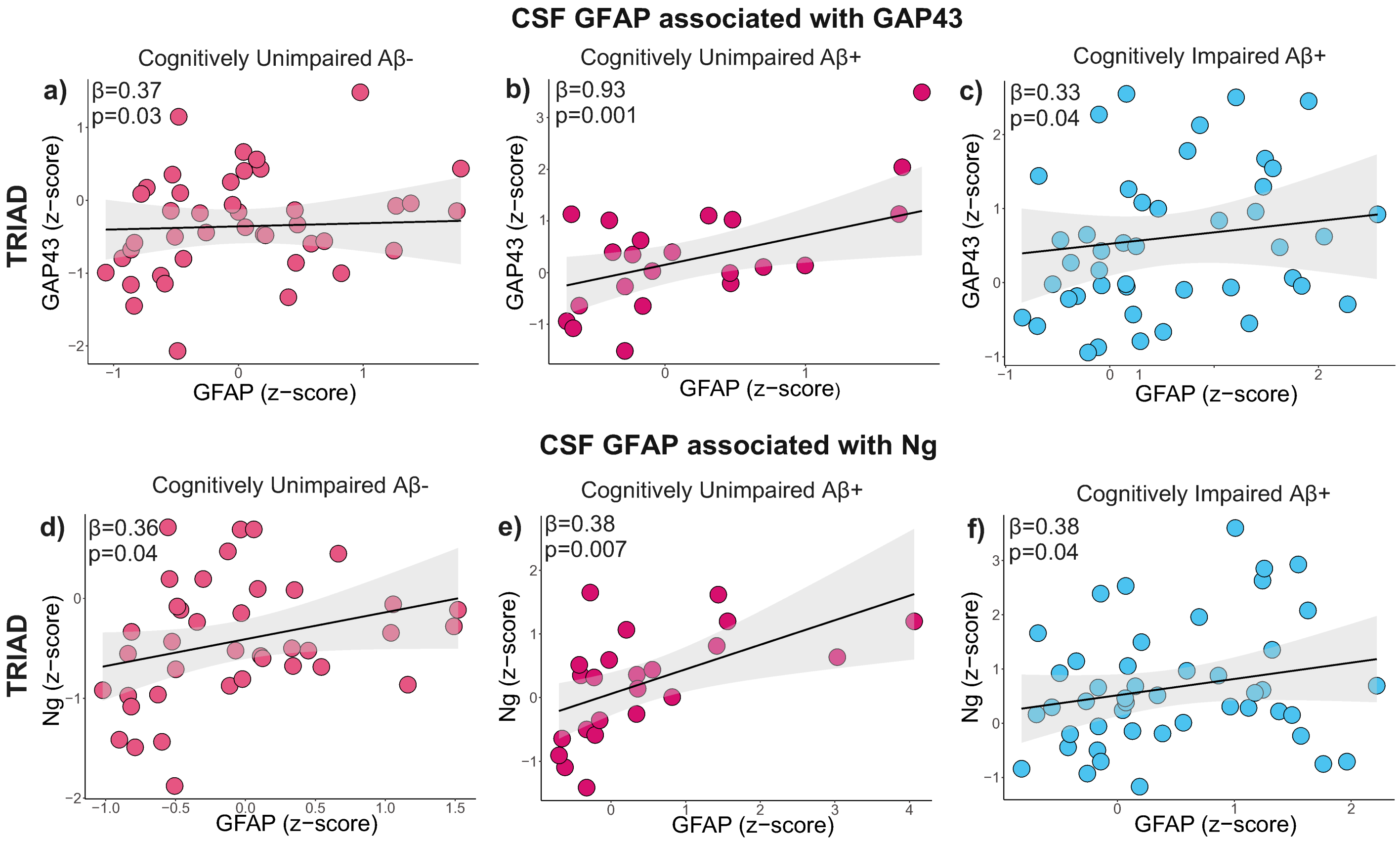

### Figure 1

**CSF GFAP is associated with synaptic dysfunction biomarkers independently of Aβ or cognitive status.** The scatter plots demonstrate a significant positive association between CSF GFAP and CSF GAP43 (**a-c**; presynaptic) and CSF Ng (**d-f**; postsynaptic) in CU Aβ-, CU Aβ+, and CI Aβ+ individuals. The linear regression models were adjusted for age and sex.

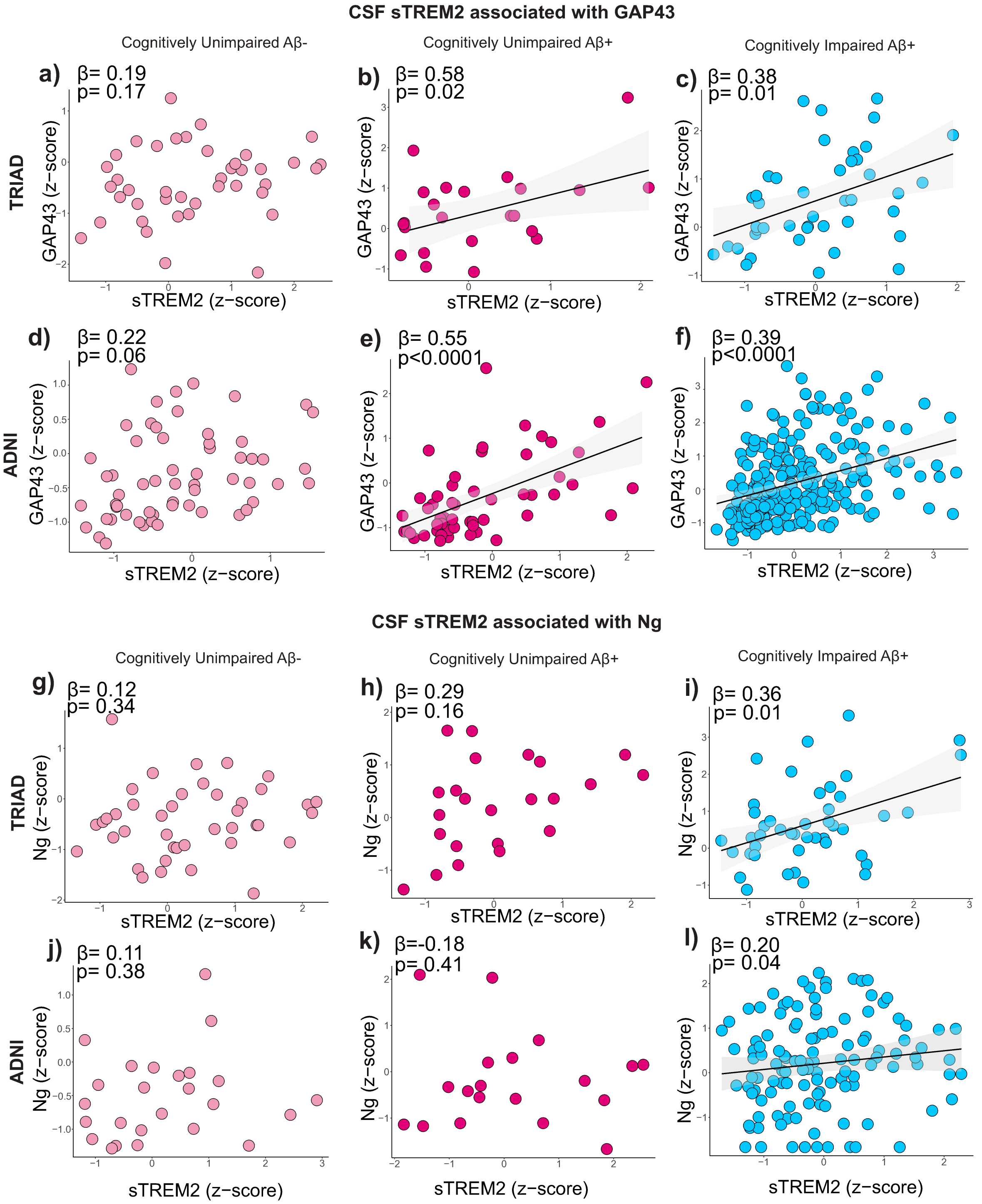

### Figure 2

**CSF sTREM2 is associated with synaptic dysfunction biomarkers only in the presence of Aβ pathology**. The scatter plots revealed that the associations between CSF sTREM2 and presynaptic marker CSF GAP43 were significant in CU Aβ+ and CI Aβ+ in TRIAD and ADNI cohorts (CU Aβ- **a,d**; CU Aβ+ **b,e**; CI Aβ+: **c,f**). CSF sTREM2 was associated with postsynaptic marker CSF Ng only in CI Aβ+ individuals in both TRIAD and ADNI cohorts (CU Aβ- **g,j**; CU Aβ+ **h,k**; CI Aβ+: **i,l**). All models were adjusted for age and sex.

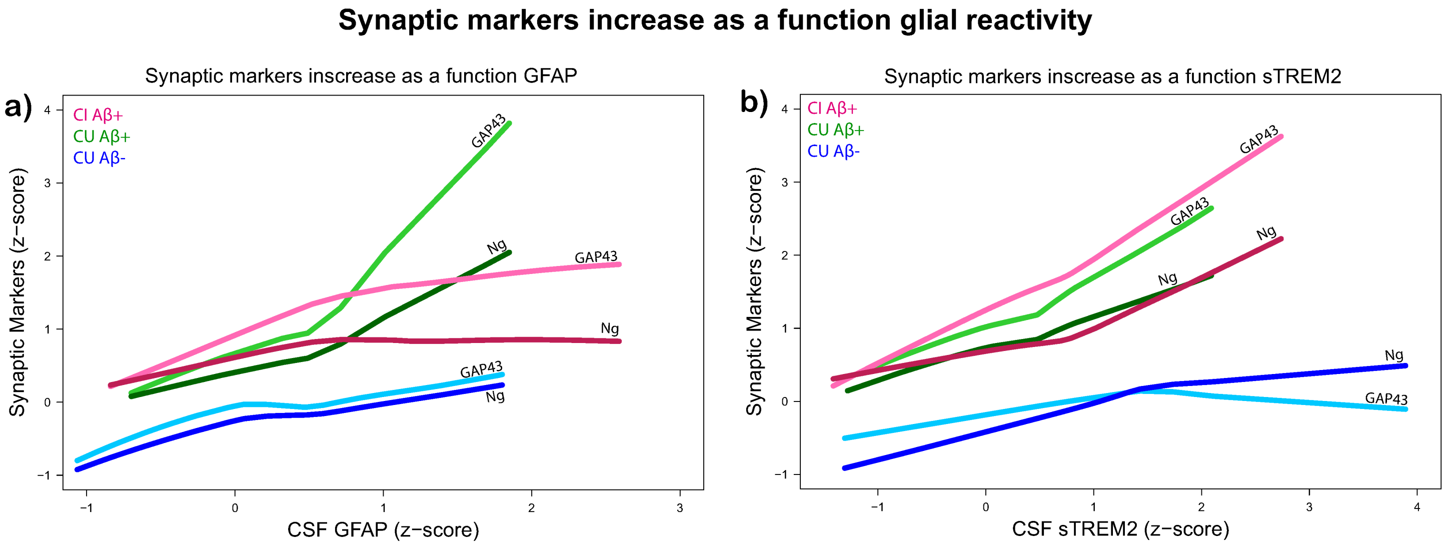

### Figure 3

**CSF synaptic biomarker levels increased as a function of CSF GFAP and CSF sTREM2.** CSF GAP43 and CSF Ng as a function of CSF GFAP (**a**) and CSF sTREM2 (**b**)**.** Lowess curves supported that synaptic dysfunction progressively increased with the increase of astrocyte reactivity biomarker levels across the aging and AD spectrum and as a function of microglial activation in the presence of Aβ pathology. Lowess curves were adjusted for age and sex.

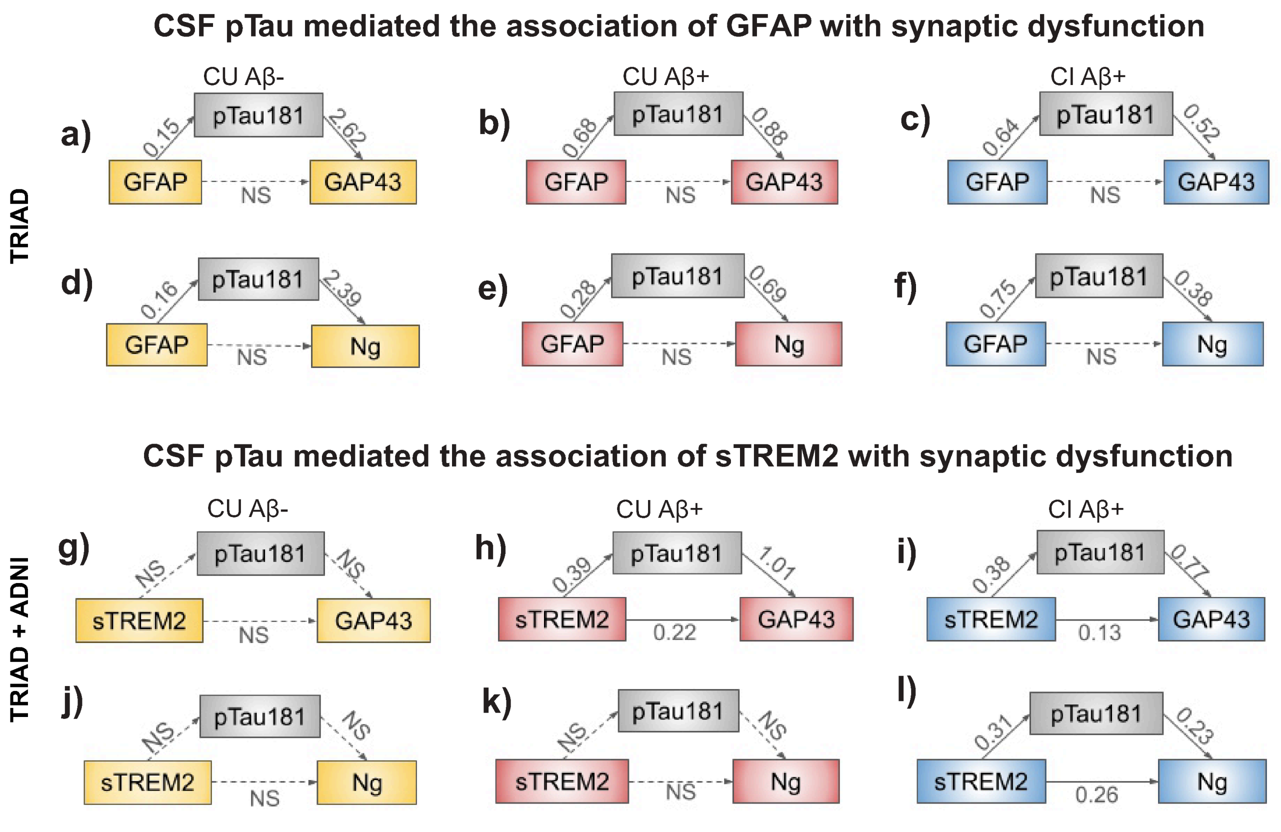

### Figure 4

**CSF pTau mediated the association of glial reactivity with synaptic dysfunction**. CSF pTau181 levels fully mediated the association between CSF GFAP on CSF GAP43 in the TRIAD cohort (Figure **a-c**) and CSF Ng (Figure **d-f**). In the TRIAD and ADNI cohorts combined, CSF pTau181 levels partially mediated the associations between CSF sTREM2 on CSF GAP43 (Figure **g-i**) and CSF Ng (Figure **j-l**), when they were present. All models were adjusted for age and sex.

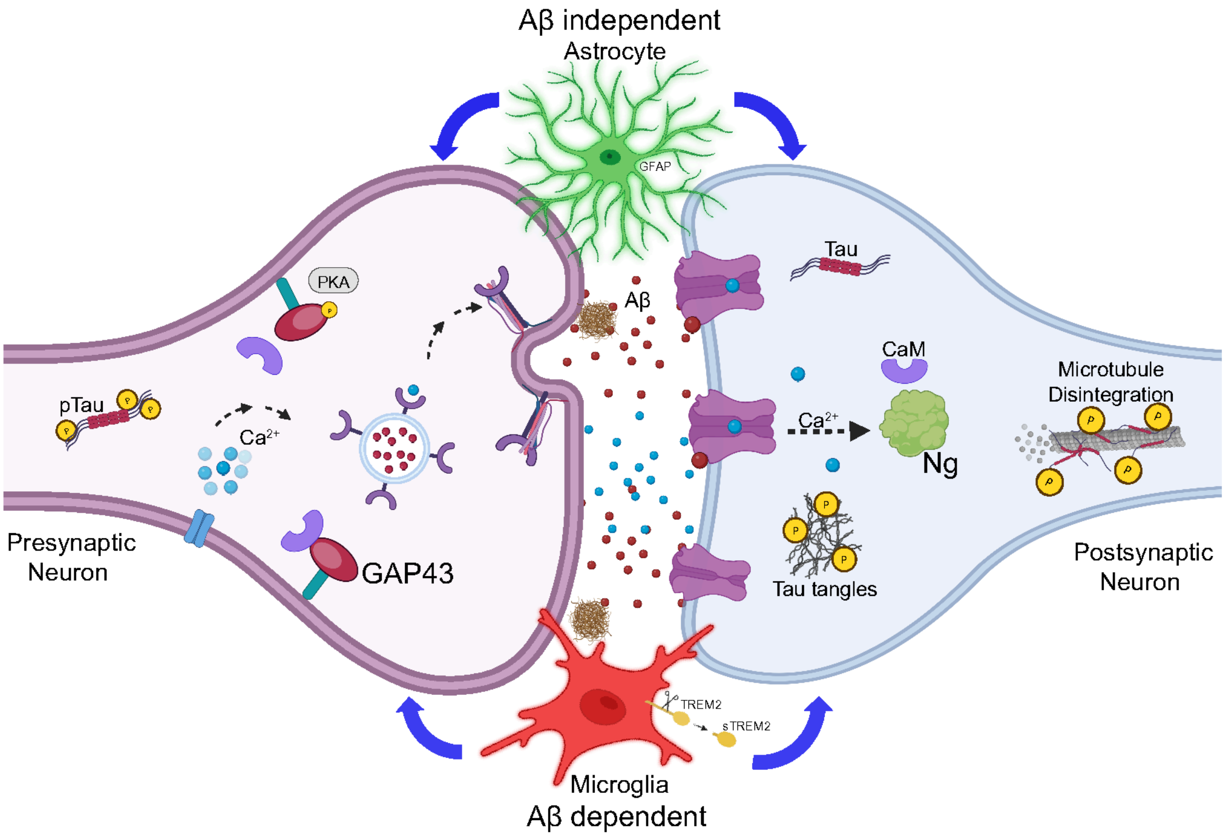

**Figure 5**

**Schematic representation of the association between astrocytic and microglial abnormalities with synaptic dysfunction.** Our study revealed that astrocyte reactivity was associated with the presynaptic (GAP43) and postsynaptic markers (Ng) independently of Aβ pathology. Conversely, microglia activation shows an association with GAP43 only in individuals Aβ+, and their association with Ng only occurs when cognitive impairment is already present.
